# Knowledge, awareness, and perception of healthcare professionals towards Long COVID in Nigeria

**DOI:** 10.3389/fpubh.2026.1768068

**Published:** 2026-06-23

**Authors:** Godspower Onavbavba, Abdulkarim Badaru, Aishat Bisoye Durojaye, Obi Peter Adigwe

**Affiliations:** National Institute for Pharmaceutical Research and Development, Abuja, Federal Capital Territory, Nigeria

**Keywords:** COVID-19, health information, post-COVID, public health, SARS-CoV-2

## Abstract

**Introduction:**

Long COVID is a complex condition with prolonged symptoms that develop after COVID-19 infection and continue beyond 12 weeks. This study aimed to assess healthcare professionals’ knowledge, awareness, and perceptions of Long COVID in Nigeria.

**Methods:**

A cross-sectional study using multistage random sampling was conducted amongst licensed healthcare professionals in Nigeria, including physicians, pharmacists, nurses, and medical laboratory scientists. A validated questionnaire was administered to practitioners in selected health facilities. Data from 405 respondents were analysed using the Statistical Package for the Social Sciences (SPSS) version 25.

**Results:**

A total of 405 valid responses were obtained, reflecting a response rate of 81%. Male and female participants were of similar proportions, as indicated by 50.1 and 49.9%, respectively. Over a quarter (26.3%) of the sample were not aware of Long COVID, and of the respondents who had heard about the condition, more than half (58.5%) indicated that they obtained their information from the internet. Participants’ understanding of Long COVID was assessed to be limited, with a mean knowledge score of 6.38 ± 2.86. As indicated by more than half (50.5%) of the study cohort, there is a lack of prioritisation in the management of post-COVID symptoms in Nigeria, as well as inadequate frameworks to mitigate the effects of the condition (45.1%). Male participants had significantly higher knowledge scores than females, with *p* = 0.006 and a standardised beta coefficient of 0.158, indicating a small-to-moderate effect size.

**Conclusion:**

The study identified knowledge gaps amongst healthcare professionals in Nigeria regarding Long COVID and provides baseline evidence to inform the development of diagnostic, preventive, and context-appropriate management strategies for the condition.

## 1 Introduction

The emergence of the COVID-19 pandemic propelled global actions towards seeking effective and safe treatments against the disease, as well as curtailing its spread. This was evidenced by the proactive measures implemented across nations and the rapid as well as unprecedented commitments to produce vaccines in less than 1 year (1, 2). In addition to this, safety guidelines such as the use of alcoholic hand sanitisers (3), wearing face masks (4), isolating suspected cases, and social distancing were implemented (5). However, despite the success of these strategies in addressing the spread of the disease, the intermittent emergence of resistant COVID-19 variants constituted a significant challenge to localised and widespread control (6).

Alongside the challenges that emerged with resistant COVID-19 variants, available evidence suggests that a proportion of the population who had been exposed to the disease experienced symptoms of Long COVID (7). This is a condition characterised by persistent long-term symptoms of the COVID-19 infection. According to a previous report (8), Long COVID can be described as a complex condition with prolonged heterogeneous symptoms that affect persons with COVID-19 and therefore requires a multidisciplinary management approach. Long COVID was identified during the first half of 2020 and gained recognition initially amongst social support groups (9). However, during this period, it received minimal attention within the healthcare space due to the global focus on more immediate challenges, such as the number of cases, hospital admissions, and mortality rates of COVID-19-infected patients (10).

Whilst the phenomenon of Long COVID, also termed Post-COVID-19 condition, is yet to be fully understood, it is often confused with COVID-19 reinfection. The condition was initially distinguished from acute SARS-CoV-2 infection by the United Kingdom guidelines, which described Long COVID as signs and symptoms that develop during or after an infection and continue beyond 12 weeks (11, 12). More recently, the World Health Organisation defined Post-COVID-19 condition as the persistence or new onset of symptoms, usually 3 months from the onset of SARS-CoV-2 infection, that last for at least 2 months and cannot be explained by an alternative diagnosis (13). A cluster of symptoms has been observed to be commonly associated with Long COVID, including fatigue, headaches, upper respiratory complaints, as well as a manifestation of multisystem challenges, such as fever and gastrointestinal tract disturbances. There is evidence that Long COVID is associated with significant economic, social, and psychological health system consequences (14). At present, the prevalence rate of the condition has been recorded to range from 2% to as high as 86% across different countries (15), and if not properly managed, can emerge as a significant public health crisis.

Despite the heightened rate of Long COVID, healthcare services specific to the condition are still evolving. There are limited specialist centres to address this condition, even in advanced countries such as the United Kingdom (16). Healthcare professionals who are the trusted sources of health-related information have also been identified to have poor attitudes towards the disease (17, 18). These factors can directly influence practices related to the condition and can lead to delayed diagnosis, poor infection control, and the spread of the disease, thereby affecting population health (19–21).

In Nigeria, Long COVID has emerged as a critical public health concern, with prevalence estimates approaching 50% in a recent rapid systematic review of African populations (22), thus necessitating urgent and comprehensive management modalities. With healthcare practitioners as the first-line professionals to respond to COVID-19 infections (23), their awareness, knowledge and perception of the condition can be harnessed to provide useful perspectives on current responses to Long COVID in the country. However, a systematic search of PubMed, Google Scholar, and African Journals Online up to December 2025 identified no prior studies in Nigeria that have robustly engaged these professionals regarding the condition. It is against this backdrop that this study aimed to determine the knowledge, awareness, and perceptions of healthcare professionals in Nigeria towards Long COVID. Findings from this study can provide insights into the current state of Long COVID management in the country as well as guide the development of initiatives and services to effectively address identified cases.

## 2 Methods

The national study adopted a cross-sectional approach to investigate the knowledge, awareness, and perception of healthcare professionals in Nigeria, including physicians, pharmacists, nurses, and medical laboratory scientists, regarding Long COVID. A well-structured questionnaire was designed for data collection.

The research instrument was developed following a comprehensive review of the literature (13, 24–26). The tool comprised relevant sections, which include the demographic data of the respondents, awareness and knowledge of Long COVID, as well as the perceptions of healthcare practitioners regarding the condition. The items assessing participants’ knowledge were answered as “true,” “false,” or “I do not know”. A five-point Likert scale was used for the section on perception as follows: 1 = Strongly disagree, 2 = Disagree, 3 = Neutral, 4 = Agree, and 5 = Strongly agree.

The questionnaire was validated by a panel of five experts involved in health research. The instrument was assessed for relevance and appropriateness. Content validity was established using the Content Validity Ratio (CVR) and Content Validity Index (CVI). The reliability of the instrument was ascertained using Cronbach’s alpha test with a value of 0.710, indicating an acceptable level of internal consistency. For individual items, CVR values ranged from 0.99 to 1.00, indicating substantial agreement among the expert panel regarding the essentiality of the items. CVI was additionally computed to complement the CVR results. Only the items that passed these tests were included in the final version. The internal consistency of the study instruments was subsequently assessed using Cronbach’s alpha. The knowledge scale (12 items) demonstrated good internal consistency (*α* = 0.861), indicating that the items measure knowledge of Long COVID. The perception scale (9 items) showed a moderate level of internal consistency (*α* = 0.691), indicating reliability suitable for exploratory analysis. The 10-item awareness scale showed acceptable internal consistency (*α* = 0.710). After accounting for missing values, there were 355 valid cases for the knowledge scale, 374 for the perception scale, and 299 for the awareness scale. The questionnaire was pretested by administration to 10 randomly selected practitioners to ensure that its structure could effectively gain the perspective of the participants in line with the aim of the study. The feedback received did not necessitate any significant change.

The minimum required sample size was calculated to be 384, which was then rounded up to 500, based on a population of approximately 940,193 workers in the health sector (27). This was computed using Epi Info software version 7 at 95% confidence level, 5% margin of error, and 50% response rate. The participants were recruited using a multistage random sampling method. One state was selected from the six geopolitical zones in the country. In each of the states included, one hospital was randomly selected, and questionnaires were issued to practitioners in the chosen facilities. The inclusion criteria for the study were healthcare providers who self-reported holding a current annual license to practice in Nigeria and who were willing to participate in the study.

Ethical approval was obtained from the National Institute for Pharmaceutical Research and Development Health Research Ethics Committee with approval number: NIPRD-HREC 25/07/2023-27, prior to the commencement of data collection. Participation in the research was voluntary, as informed consent was obtained from the respondents before the administration of the questionnaires.

Following the collection of data, analysis was carried out using Statistical Package for Social Sciences (SPSS) version 25. Both descriptive and inferential statistical analyses were undertaken. For the knowledge section, each correct response was assigned a score of 1, whilst incorrect responses and ‘I do not know’ responses were assigned a score of 0, giving a maximum possible score of 12. The total knowledge score of each respondent was aggregated using Bloom’s cut-off standard of categorisation.

The questionnaire comprised 12 knowledge items; the percentage thresholds were converted into corresponding integer score ranges. Scores of 0–7 (≤ 59%) were categorised as poor knowledge, 8–9 (60–79%) as average knowledge, and 10–12 (≥ 80%) as good knowledge. Inferential statistical analysis was then undertaken to determine the association between the responses of the participants and their demographic characteristics. A *p-*value of 0.05 or less was used as a threshold for statistical significance. Data were handled using listwise deletion to account for incomplete questionnaire sections.

Knowledge scores were analysed as a continuous composite variable. Parametric tests, including independent sample *t*-tests, one-way ANOVA, and multiple linear regression, were employed based on the robustness of the sample size. Homogeneity of variance was verified using Levene’s test, with the significance threshold set at *p* ≤ 0.05.

## 3 Results

### 3.1 Socio-demography

A total of 405 valid responses were obtained from the 500 questionnaires distributed, giving a response rate of 81%. Male and female participants were of a similar proportion, as represented by 50.1 and 49.9%, respectively. Respondents between the ages of 18 and 30 years constituted about two-thirds (63.6%) of the study cohort. With respect to educational attainment, three-quarters (74.4%) of the sample had a first degree. Other details are presented in Table 1.

**Table 1:** Socio-demographic characteristics.

Variables	Frequency (%)
Gender	
Male	203 (50.1)
Female	202 (49.9)
Age	
18–30	253 (63.6)
31–40	86 (21.6)
41–50	56 (14.1)
>50	3 (0.8)
Highest educational level	
Diploma	50 (12.9)
First degree	288 (74.4)
Master’s degree	42 (10.9)
Doctorate degree	7 (1.8)
Number of years in practice	
<5	251 (63.5)
5–10	72 (18.2)
11–15	33 (8.4)
>15	39 (9.9)
Sector	
Government sector	281 (74.5)
Private sector	89 (23.6)
Development partners	6 (1.6)
Others	1 (0.3)

### 3.2 Awareness and knowledge about Long COVID

About three-quarters of the participants (73.7%) disclosed that they were aware of the existence of Long COVID. A quarter (26.3%) of them indicated that they had not heard about the condition prior to the research.

With respect to sources of information, over half (58.5%) of the respondents indicated ‘internet’ as their means of knowledge about Long COVID, and this was closely followed by social media (43.2%).

**Figure 1:**
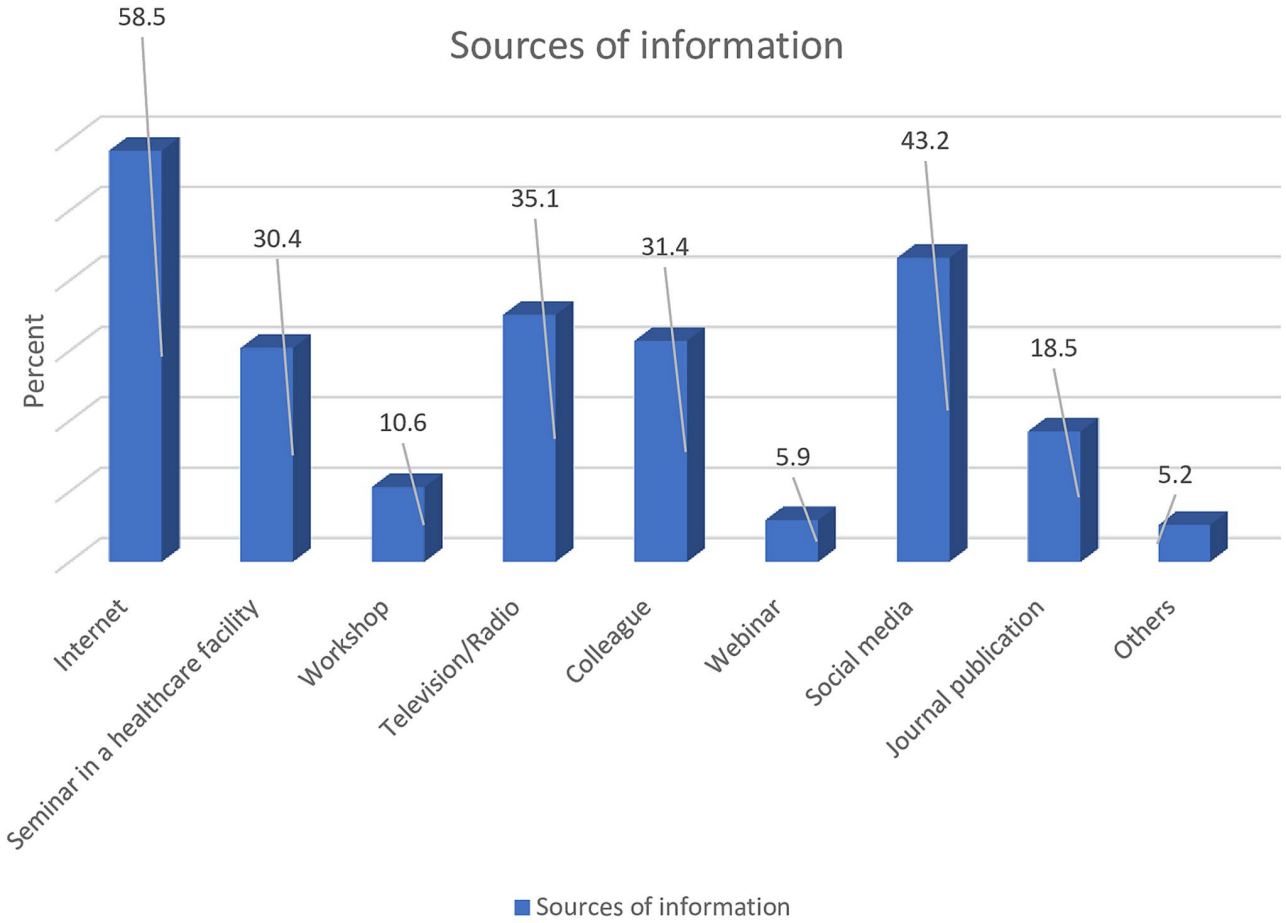
Sources of information about Long COVID.

Furthermore, about a third (30.4%) of the sample indicated that information regarding the condition was obtained from seminars, and 31.4% reported the source of their awareness to be from colleagues. Further details are presented in Figure 1.

### 3.3 Knowledge of healthcare practitioners towards Long COVID

From the participants’ assessment, the maximum obtainable knowledge score was 12, and the overall mean was 6.38 ± 2.86. Based on Bloom’s standard of categorisation, only 11.1% had a good knowledge of Long COVID. Over a quarter (27.5%) of the participants reported average knowledge, and nearly two-thirds (64.4%) of the sample had poor knowledge regarding the condition.

Three-quarters (74.1%) of the participants, however, answered correctly that Long COVID was the continuation of COVID-19 symptoms following recovery, and about half (47.4%) indicated that symptoms may need to persist for 2 months before they can be classified as such. Although a third (31.6%) of the participants appropriately noted the non-existence of definite treatments for the condition, more than two-thirds (69.4%) were knowledgeable about the use of COVID-19 vaccines to reduce the risk of Long COVID. Other relevant details about the participants’ knowledge of the condition are presented in Table 2.

**Table 2:** Knowledge of healthcare practitioners about Long COVID (Correct responses).

SN	Statement	Frequency (%)
1	Long COVID is a condition where individuals continue to experience symptoms after recovering from an acute COVID-19 infection.	300 (74.1)
2	Fatigue is a common symptom of Long COVID.	287 (70.9)
3	Respiratory symptoms, such as shortness of breath and chest pain, can be present in individuals with Long COVID.	319 (78.8)
4	Long COVID can cause bodily aches such as muscle aches, headaches, and joint pains.	255 (63.0)
5	Symptoms must persist for 2 months before they can be considered as those of Long COVID.	192 (47.4)
6	Long COVID can be easily transmitted.	77 (19.0)
7	Vaccination reduces the risk of developing long COVID.	281 (69.4)
8	Long COVID may affect multiple organs in the body.	278 (68.6)
9	The most commonly reported symptoms of Long COVID are fatigue and memory problems.	165 (40.7)
10	Definite treatment exists for Long COVID.	128 (31.6)
11	Long COVID is a condition that affects only individuals with severe cases of COVID-19 during the acute phase.	118 (29.1)
12	All patients present with the same pattern of symptoms.	180 (44.4)

### 3.4 Preparedness for management of Long COVID

**Figure 2:**
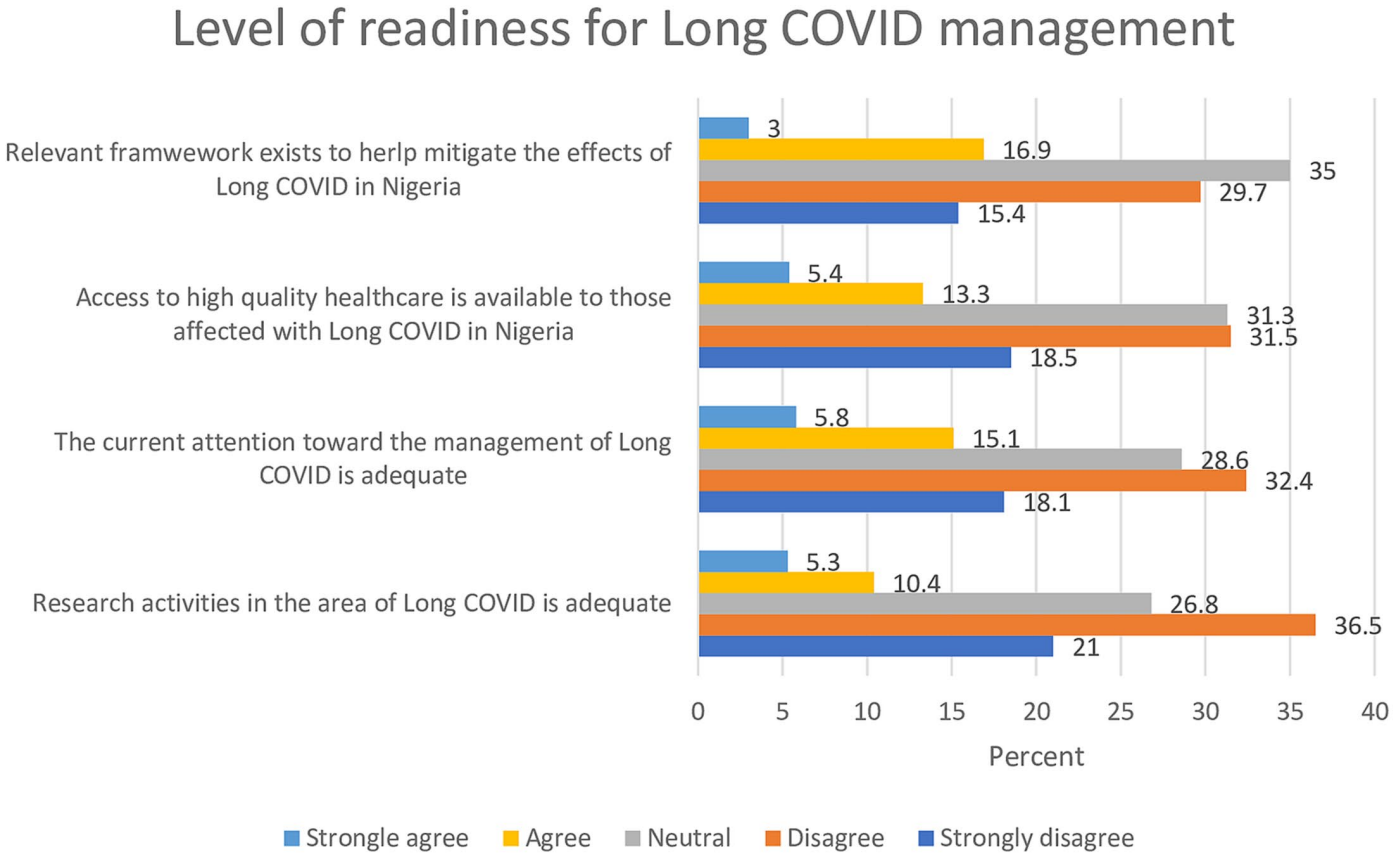
Level of readiness for Long COVID management.

More than half (50.5%) of the study cohort indicated that there was inadequate attention towards the management of Long COVID symptoms in Nigeria. A similar proportion (50%) further highlighted that quality healthcare for the affected survivors was suboptimal, whilst a third (31.3%) of them had a neutral disposition to this. Close to half (45.1%) reported the absence of relevant frameworks to mitigate the effects of Long COVID in Nigeria, and 57.5% indicated that research and scientific inquiry in this area had been insufficient. Figure 2 provides more information on the level of readiness to manage Long COVID in the country.

### 3.5 Effects of Long COVID

**Figure 3:**
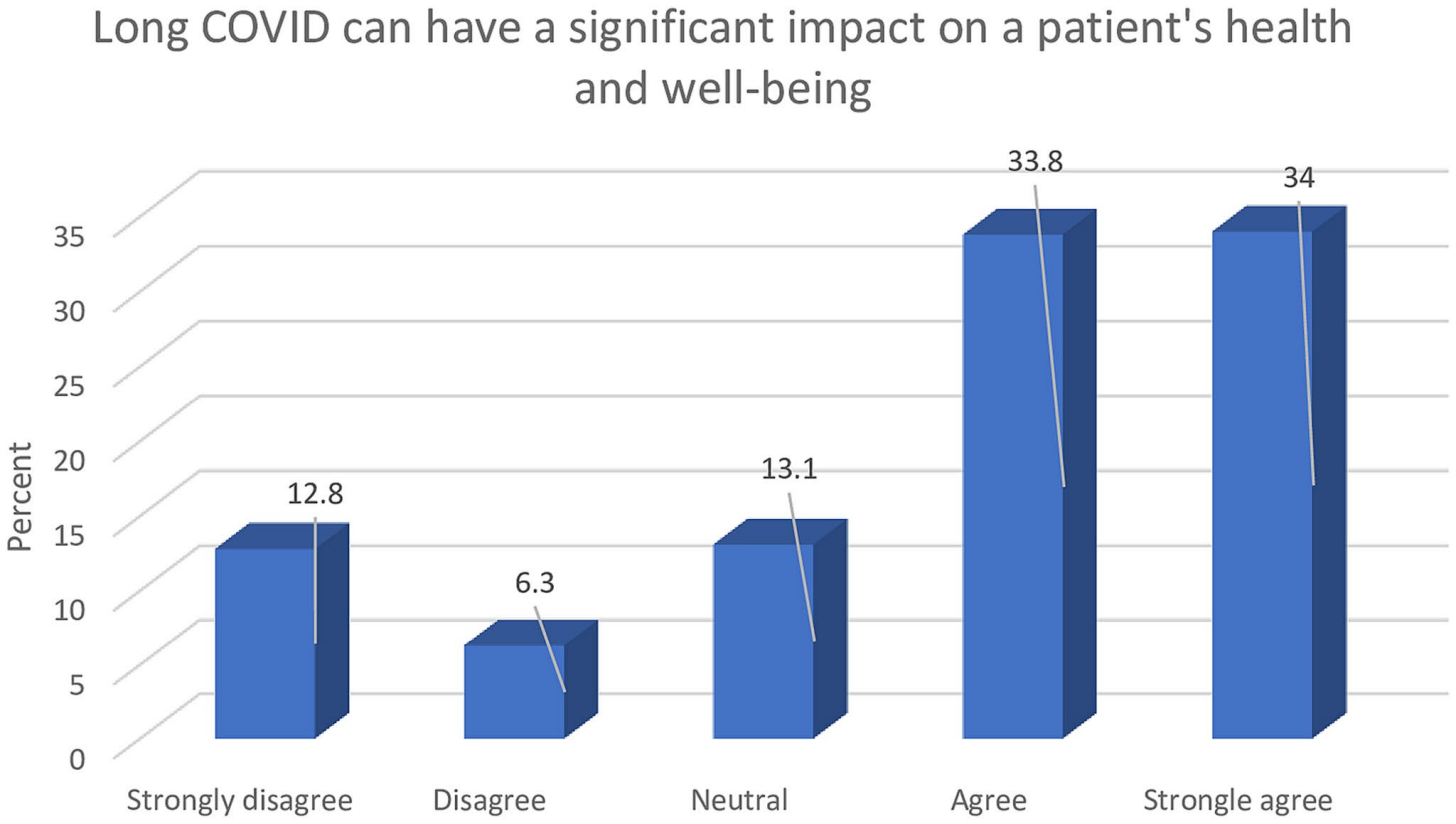
Impacts of Long COVID on patients’ well-being.

With respect to the effects of Long COVID, over two-thirds (67.8%) of the practitioners agreed on the potential of the condition to significantly affect the well-being and health of patients. Further information regarding this is presented in Figure 3.

One-fifth (19.1%) of the study respondents indicated that Long COVID had no significant impact on patients.

### 3.6 Improving care for Long COVID

**Figure 4:**
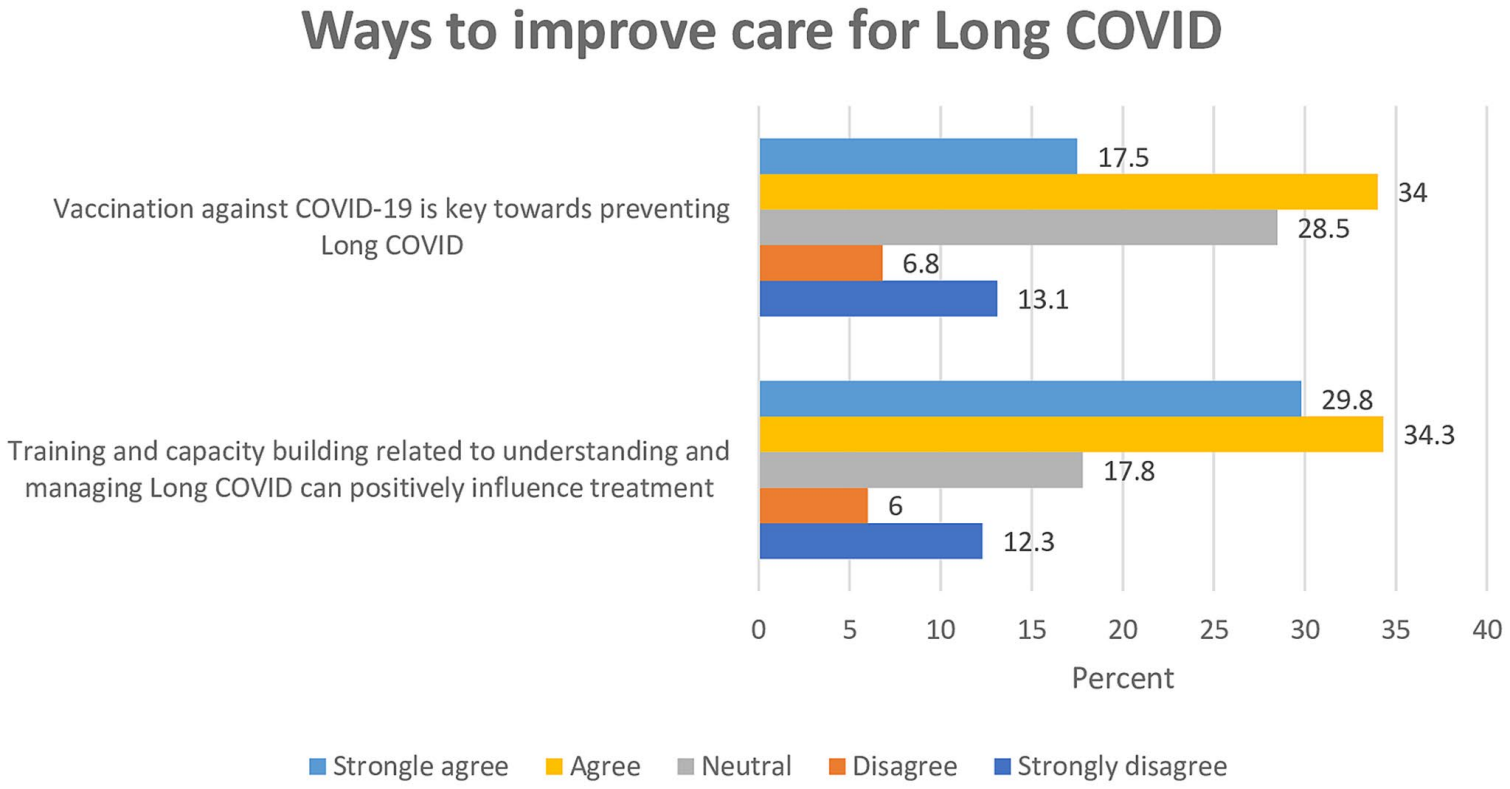
Ways to improve care for Long COVID.

Two-thirds (64.1%) of the participants agreed that the management of Long COVID can be improved through training and capacity-building of healthcare practitioners More than half (51.3%) identified COVID-19 vaccination as key to prevention, whilst approximately a fifth (19.9%) disagreed with this position. Further information on the ways to improve care for Long COVID is presented in Figure 4.

More than half of the respondents (51.3%) indicated that vaccination against COVID-19 was key to preventing Long COVID; however, about a fifth of them (19.9%) opposed this position.

### 3.7 Relationship between the socio-demographic characteristics of participants and their level of knowledge regarding Long COVID

Bivariate analysis revealed a significant relationship between the gender of the participants and their mean knowledge score. The male participants had a higher mean knowledge score than the female respondents (*p* = 0.005). One-way ANOVA revealed no statistically significant differences in total knowledge scores across age (*F*(4, 347) = 0.055, *p* = 0.679), profession and other socio-demographic variables examined, with all *p*-values exceeding 0.05. The details of the analysis are presented in Table 3.

**Table 3:** Relationship between the socio-demographic characteristics of participants and their level of knowledge regarding Long COVID.

Variable	Mean ± SD	Test of significance (*p*)
Gender		*t* = 8.012 (0.005)
Male	6.78 ± 2.71	
Female	5.98 ± 2.96	
Age (years)		*F* = 0.505 (0.679)
18–30	6.45 ± 2.80	
31–40	6.55 ± 2.73	
41–50	6.27 ± 3.16	
Above 50	4.67 ± 1.53	
Highest educational qualification		*F* = 0.859 (0.462)
Diploma	6.08 ± 2.31	
First degree	6.45 ± 2.92	
Master’s degree	6.36 ± 2.91	
Doctorate degree	7.86 ± 1.95	
Years of practice		*F* = 1.140 (0.333)
Less than 5	6.43 ± 2.84	
5–10	5.94 ± 2.97	
11–15	6.94 ± 2.70	
Above 15	6.69 ± 2.87	
Sector of practice		*F* = 0.386 (0.763)
Government sector	6.43 ± 2.94	
Private sector	6.42 ± 2.49	
Development sector	7.67 ± 3.39	
Others	7.00 ± 0.000	

Furthermore, a multiple linear regression analysis with 95% CI was conducted to identify independent predictors of knowledge scores, adjusting for gender, age, profession, educational level, years of practice, and sector. Gender remained the only statistically significant independent predictor (*B* = 2.362, SE = 0.851, *β* = 0.158, *t* = 2.777, *p* = 0.006), indicating that male participants had higher knowledge scores compared to female participants. Age (*p* = 0.469), profession (*p* = 0.467), educational level (*p* = 0.518), years of practice (*p* = 0.275), and sector (*p* = 0.758) were not significantly associated with knowledge scores (Table 4).

**Table 4:** Multiple linear regression analysis of factors associated with knowledge score.

Variable	*B*	SE	*β*	*t*	*p*-value
Constant	17.769	2.797	–	6.353	<0.001
Gender	2.362	0.851	0.158	2.777	0.006
Age	0.558	0.770	0.054	0.725	0.469
Profession	−0.314	0.430	−0.043	−0.729	0.467
Education	0.603	0.932	0.041	0.646	0.518
Years of practice	−0.698	0.639	−0.088	−1.093	0.275
Sector	−0.262	0.849	−0.018	−0.309	0.758

*B*, unstandardised coefficient; SE, standard error; *β*, standardised coefficient; *p*-value indicates statistical significance at *p* < 0.05.

## 4 Discussion

The findings from this study revealed that there was significant awareness amongst healthcare professionals about Long COVID. However, regardless of their age, educational qualification, or years of experience, this knowledge was inadequate. The health professionals had limited understanding of Long COVID symptom patterns and available treatments. This is contrary to reports from Bangladesh, where most practitioners surveyed demonstrated good or excellent knowledge about the condition (25). Although male respondents in this study had a higher mean knowledge than their female counterparts, the overall limitation in their understanding of Long COVID may have stemmed from the relatively new concept of the condition and its underestimated incidence in this setting (28, 29). With the identified gaps in the understanding of health practitioners in Nigeria on the treatment and symptomatic patterns of the condition, training professionals on Long COVID has become important. This can be done through seminars, workshops, and continuing professional development programmes. These are critical capacity building tools for health sector interventions, especially those that are new to a contextual setting. Training modalities can be tailored to fit the scope of each profession and be made available to practitioners across the primary, secondary, and tertiary healthcare facilities in the country.

The limited knowledge amongst healthcare practitioners in Nigeria regarding Long COVID can be further attributed to the inadequate scientific evidence available to influence the awareness of practitioners regarding the condition. As observed from the responses, the major sources of information about Long COVID were the internet and social media, rather than evidence-based literature such as peer-reviewed journal publications and output from science-inclined seminars and workshops. Whilst outsourcing health information from social media platforms can be useful for gaining awareness about diseases (30), the reliance on internet and social media as information sources may reflect variability in access to formal scientific updates; however, this study did not examine the relationship between information sources and knowledge scores, and no causal inference can be made. The participants identified inadequate research on Long COVID as a major constraint to evidence-based practice, and this buttresses the importance of promoting scientific inquiries regarding the condition. These measures can help to enhance the level of awareness, knowledge and perception of healthcare practitioners regarding Long COVID, thus promoting appropriate diagnostic and management modalities.

The study further revealed perceived gaps in policy and practice readiness for the management of Long COVID in Nigeria, based on respondents’ views. Participants indicated that no relevant framework exists to attenuate the effects of Long COVID, buttressing the non-availability of high-quality care for those affected by the condition. In some settings, self-management strategies and specialised centres have been reported; however, differences in health-system capacity, resources, and stage of service development limit direct comparison with the Nigerian context (15, 31). These findings therefore suggest the need for further policy and service-level evaluation, and relevant frameworks to address the repercussive effects associated with COVID-19 and other future health emergencies in the country.

Novel insights from this study also linked overall management of Long COVID in Nigeria with the implementation of contextual immunisation strategies. Respondents perceived vaccination as an important preventive measure, although current evidence suggests that vaccination may reduce but does not eliminate the risk of Long COVID. This is in line with similar studies, which reported that improving the immunity of the population against COVID-19 is associated with a low risk of developing persistent post-COVID symptoms (32–34). However, available evidence suggests that this may be a challenge for the Nigerian setting, given the prevalence of vaccine hesitancy in the country (1, 35, 36). Therefore, as part of the means to protect patients against Long COVID, the sensitisation of the citizens towards immunisation uptake is of critical importance. It is also imperative to ensure that the prevailing factors that affect the willingness of the public to accept vaccines are adequately addressed.

In addition to vaccination, the study identified the importance of training and capacity building of healthcare professionals as a means of improving the management of Long COVID. Successful implementation of this strategy may, however, be influenced by system-wide availability of data on the diagnosis and other relevant aspects of treatment and management of the condition. The lack of adequate data, stemming from the paucity of research activities in this thematic area, can pose a critical challenge to informed and quality care towards Long COVID for patients (37). These and other far-reaching consequences can further significantly impact patient health and well-being (38). It is therefore imperative to leverage lessons learnt during COVID-19 to also institutionalise research, training, and other evidence-generating and utilisation activities for Long COVID-related policies and practices. This will consequently enhance public health outcomes, as well as contribute to the overarching objective of reducing the disease burden in the country.

This study has some limitations that should be considered when interpreting the findings. The cross-sectional design limits the ability to establish causal relationships, and the use of a self-reported questionnaire introduces the possibility of bias. Furthermore, responses were based on participants’ perceptions, which may not fully reflect actual policy, service availability, or clinical practice in the country. Despite these limitations, the study provides valuable baseline evidence on healthcare professionals’ knowledge, awareness, and perceptions of Long COVID across Nigeria’s diverse geopolitical zones.

## 5 Conclusion

This study provides novel insights into the perspectives of healthcare professionals in Nigeria regarding Long COVID. It emerged that amongst practitioners, there were significant knowledge limitations regarding the condition, regardless of age, educational qualifications, or years of practice. Findings also highlighted limited research and related evidence-generating activities on Long COVID, hence, the inadequate attention to patient care revealed in the study setting.

The identified gaps in this study underscore the urgent need for contextual research, as well as Long COVID-related capacity building and continuous professional development. In addition to the relevant technical aspects, training should include values, attitudes, skills, and knowledge aligned with professional socialisation ideals. There is also a need for the development of proactive legislation, policies, and practice frameworks that comprehensively address long-term effects of not just COVID-19, but also other future potential health emergencies. This will directly reduce disease burden in the country, whilst also enabling a rapid system-wide capacitation of healthcare practitioners and other key stakeholders.

Other novel findings that emerged from the study include important population-wide strategies. For instance, citizen self-management using tools like vaccination, which was identified as a critical means for self-protection against Long COVID. Evidence-based sensitisation programmes were also highlighted as critical for addressing prevailing factors that reduce public willingness to accept vaccines. These initiatives can not only improve management of Long COVID in Nigeria, they can also significantly strengthen other response mechanisms within the overarching system.

## Data Availability

The raw data supporting the conclusions of this article will be made available by the authors, without undue reservation.
